# Psychological Resources and New Product Development Team Adaptability: A Moderated Mediation Model

**DOI:** 10.1177/10464964241283695

**Published:** 2024-09-27

**Authors:** Dominic L. Marques, Caroline Aubé, Vincent Rousseau, Eric Brunelle

**Affiliations:** 1 HEC Montreal, QC, Canada; 2 University of Montreal, QC, Canada

**Keywords:** psychological capital, team adaptability, new product development, team creativity, outcome focus

## Abstract

Despite the importance of adaptability for new product development team performance, empirical research that explores the factors promoting their adaptive capacity is limited. This study draws from psychological resource theories to examine how and under which conditions team psychological capital enhances the adaptivity of new product development teams. Data were collected from 198 student teams who took part in a project management simulation. Results showed that the positive relationship between team PsyCap and team adaptivity travels through team creativity and that the level of outcome focus moderates the first stage of this relationship.

In the current global economy, the organizational capacity to develop unique and compelling products is a critical determinant of firm performance (Cheng & Yang, 2019). In order to facilitate this process, many organizations rely on new product development (NPD) teams (Zhang & Min, 2019). The rationale behind this collective approach to NPD is that through teamwork, members are able to share knowledge and integrate their functional expertise, which results in the generation of creative ideas and innovative products. However, research shows that NPD projects have high failure rates (Cooper, 2019). To explain this situation, researchers have pointed out the shortcomings of NPD teams in terms of adapting to high levels of uncertainty. Specifically, NPD teams have to deal with unstable specs and requirements, fluid team boundaries and membership, changing market forces, and technological advances, which all render team performance more challenging (Stock et al., 2021). To manage these types of contingencies and to ultimately reach their objectives, NPD teams must therefore possess strong adaptive capacities (Cooper, 2019).

Despite the recognition that adaptability lies at the heart of NPD team performance, empirical studies that investigate the factors promoting their adaptative capacity remain scarce, highlighting a limited understanding of the phenomena (Durmusoglu & Calantone, 2022). This shortcoming is representative of the state of affairs in the broader team literature where there is also a limited number of studies that empirically examine the inputs and processes that contribute to team adaptation (Christian et al., 2017; Maynard et al., 2015). Therefore, the overarching objective of the present study is to explore team-level factors that promote the adaptability of NPD teams. More precisely, based on previous research showing that psychological aspects play a pivotal role in the context of NPD teams (Sivasubramaniam et al., 2012) and that psychological resources are important drivers of successful adaptation (Hobfoll, 2002; Van den Heuvel et al., 2014), we draw from psychological resource theories (e.g., Bandura & Wessels, 1997; Luthans & Youssef, 2004) to propose that team psychological capital (team PsyCap) positively influences the adaptivity of NPD teams.

The construct of psychological capital (PsyCap) refers to a positive psychological state characterized by the possession of four key resources: optimism, self-efficacy, resilience, and hope (Luthans et al., 2007). At its core, PsyCap consists of affective and cognitive components that relate to an individual’s beliefs about his or her capacities and chances of success on a certain task or in a specific context (Stajkovic, 2006). Although hope, self-efficacy, resilience, and optimism are all valid psychological constructs and have often been studied in a standalone manner, psychological resource theories and more specifically the notion of resource caravan explain that psychological resources usually overlap, covary, and travel together and are thus often better understood as manifestations of broader psychological capacities (Hobfoll, 2002; Luthans et al., 2007). On that matter, empirical studies provide consistent evidence that the four psychological resources that compose team PsyCap consistently load onto a single higher-order construct (Avey et al., 2009; Luthans et al., 2007; Stajkovic, 2006). In addition, previous research has also validated the state-like nature of PsyCap and its discriminant validity from other more stable individual difference variables such as intelligence, core self-evaluations, and personality traits (Avey et al., 2009; Luthans et al., 2007).

At the team level, PsyCap is considered as an emergent psychological state and as a shared team property (B. J. West et al., 2009). This conceptualization is consistent with previous research in social psychology which explained that when a group of people work together with frequent interactions, shared objectives and experiences, and clear boundaries, they tend to converge in their psychological states and develop shared psychological resources (Mathieu et al., 2000). Also, team PsyCap is an isomorphic representation of PsyCap (Heled et al., 2016). As such, team PsyCap is structurally equivalent to individual PsyCap in that the four psychological resources of hope, self-efficacy, resilience, and optimism keep the same nature and meaning at the team level. However, the elevation to the team results in a conceptually distinct construct (Dawkins et al., 2015). Indeed, as a team property, team PsyCap resides at a higher level than individual PsyCap and refers to a different target of evaluation. Whereas team PsyCap captures the evaluations that members make about the psychological resources of their team, PsyCap refers to the appraisal that individuals make about their own levels of hope, self-efficacy, resilience, and optimism.

The second objective of the present study is to clarify the behavioral pathway that underlies the relationship between team PsyCap and NPD team adaptivity. Specifically, considering that psychological resources provide the motivation and willpower to engage and persist in creative processes (Sweetman et al., 2011; Tierney & Farmer, 2002), and that positive psychological states have been found to broaden cognitive capabilities (Fredrickson, 2001; George & King, 2007), this study investigates the mediating role of team creativity in the relationship between team PsyCap and NPD team adaptivity.

Lastly, previous research suggests that facets of the team climate such as team goal orientations can influence the extent to which teams exhibit their psychological attributes to engage in creative processes (Cai et al., 2019; Gong et al., 2013). Team goal orientations refer to a shared understanding between members of the way to approach tasks and of the types of results to prioritize (Gong et al., 2013). As such, team goal orientations send situational and conforming cues to members that influence the types of behaviors and practices they engage in. Considering that the creative and adaptive benefits of a team performance and outcome orientation are still not well understood (Caniëls et al., 2019; Christian et al., 2017), the third objective of this study is to investigate the moderating effect of outcome focus on the indirect relationship between team PsyCap and NPD team adaptivity. More precisely, we argue that a high degree of outcome focus will exert a negative influence on the relationship between NPD team PsyCap and team creativity. Taken together, these hypotheses specify a moderated mediation model in which team PsyCap exerts a positive indirect effect on NPD team adaptivity that travels through team creativity, and in which the level of outcome focus moderates the first stage of this relationship (see Figure 1). By testing such a model, this study provides a fine-grained analysis of how and under which conditions shared psychological resources promote the adaptive capacities of NPD teams. In doing so, the present study advances understanding of the factors that enhance the adaptability of NPD teams.

**Figure 1.**
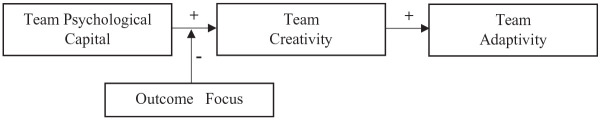
Hypothesized research model.

## Theoretical Background and Hypotheses

### Psychological Resource Theories

As a subset of broader resource theories, psychological resource theories are concerned with internal characteristics and psychological strengths, as well as the ways in which possession of these personal resources contributes to optimal functioning across multiple life domains and professional settings (Gorgievski et al., 2011; Hobfoll, 2002). More precisely, psychological resource theories highlight that psychological resources such as hope, self-efficacy, resilience, and optimism are important drivers of successful adaptation (Van den Heuvel et al., 2010). Indeed, these theories point to two critical roles played by psychological resources in the adaptation process. First, change and adaptation imply a state of disequilibrium and subsequent adjustments that can be demanding and stressful (Vakola & Nikolaou, 2005). On that matter, research has shown that psychological resources often reduce the strains and stresses associated with adaptive demands (Hobfoll, 2002). This means that individuals who have access to a deeper pool of psychological resources are better able to withstand and cope with the negative effects of change, which makes them more likely to adapt successfully. Second, psychological resources provide a stream of physical and mental energy that allow individuals to engage in behaviors that promote adaptation (Van den Heuvel et al., 2014). In other words, individuals who possess a wide array of psychological resources are more likely to adopt adaptive behaviors in the face of change. At the team level, this notion suggests that collective hope, efficacy, resilience, and optimism carry important adaptive benefits that can be explained by the types of behaviors members engage in. This is consistent with the Input–Throughput–Output model of team adaptation (Burke et al., 2006), which explains that adaptation inputs such as team resources influence adaptive processes that, in turn, affect team adaptive performance. In other words, team member behaviors are considered important explanatory mechanisms in the association between shared psychological resources and team adaptability. Therefore, in the context of NPD teams, we argue that team creativity will mediate the positive relationship between team PsyCap and team adaptivity.

### Team Psychological Capital and Team Creativity

Team creativity is defined as the extent to which team members generate a wide array of novel and useful ideas (Amabile & Pratt, 2016; M. A. West, 2002). In that way, team creativity refers to the collective process of idea generation and to the relative value and originality of those ideas. Drawing from previous research showing that positive psychological resources are crucial for creativity (N. Anderson et al., 2014; Tierney & Farmer, 2002), we hypothesize that team PsyCap will exert a positive influence on the creativity of NPD teams.

Previous research highlights two reasons shared positive psychological states and resources have the potential to promote the creativity of NPD teams. On the one hand, creativity is considered a cognitively complex and demanding process (Amabile, 1983). As such, the collective engagement in creative processes requires a motivational force to persevere and move beyond the challenges inherent in creative work (Tierney & Farmer, 2002). On that matter, previous research indicates that the four psychological resources composing team PsyCap can facilitate this motivational process. First, hope, which is defined as a motivational state that is based on a sense of agency and goal-directed energy, has been found to generate the willpower to engage in demanding activities and to pursue difficult goals (Snyder et al., 1991). Importantly, hope has been shown to promote the engagement and persistence in creative processes (Sweetman et al., 2011). Similarly, efficacy beliefs have also been identified as important factors in the adoption of creative behaviors (Gupta & Singh, 2014). At the team-level, collective efficacy refers to members’ beliefs about the capacities and chances of success of their team (Bandura & Wessels, 1997). Teams with high levels of collective efficacy are confident that they possess the abilities and skills needed to successfully execute a specific task within a given context (Stajkovic & Luthans, 1998). This suggests that NPD teams with high levels of collective efficacy will be more confident and therefore more likely to engage in creative behaviors (Tierney & Farmer, 2002). Lastly, because the creative process is filled with repeated iterations, setbacks, and failures (Amabile & Pratt, 2016), resilience and optimism are also considered important psychological resources for creativity (Luthans et al., 2007; Sweetman et al., 2011). Indeed, resilience, which is defined as the capacity to cope successfully and bounce back quickly from adversity, uncertainty, and failure (Luthans, 2002), has been recognized as key in overcoming the challenges inherent in creative work and persevering in the adoption of creative behaviors (Luthans et al., 2007). Optimism, which broadly refers to a generalized positive outlook characterized by positive expectancies and internal attributions of success (Luthans, 2002), plays a similar role as resilience in that it provides the confidence and motivation needed to deal effectively with the setbacks and failures inherent in creativity (Sweetman et al., 2011).

The second potential reason relies on the idea that shared psychological resources can promote the collective engagement in creative processes by broadening the cognitive scope of team members (George & King, 2007). In other words, when team members possess shared positive psychological resources, they will tend to develop broader and more flexible cognitive structures which, in turn, will promote their collective capacity to engage in creative processes. For example, hope has been identified as a psychological resource that promotes pathway thinking (Snyder, 2000). Pathway thinking represents the ability to generate multiple pathways to goal attainment (Snyder, 2000). As such, high hope fosters the exploration of alternatives, unconventional thinking, and creative problem-solving (Sweetman et al., 2011). This entails that NPD teams with high levels of hope will tend to be cognitively more flexible and thus better able to come up with alternative ways to approach situations and solve problems, which is a critical aspect of creativity (Amabile & Pratt, 2016). Moreover, efficacy beliefs have also been shown to broaden cognitive processes. For instance, efficacy beliefs have been linked to wider information searches and to higher levels of inventiveness and resourcefulness, which are also important aspects of creativity (Tierney & Farmer, 2002). This means that when NPD teams are confident in their collective abilities and chances of success, they are more likely to delve into unknown areas to propose new and useful ideas.

All in all, by generating the collective willpower to engage in creative behaviors and by broadening the cognitive scope of team members, team PsyCap provides the motivation and cognitive capacity required to engage in creative behaviors. On the basis of this assessment, we formulate our first research hypothesis:

*H1*: Team psychological capital is positively related to NPD team creativity.

### Team Creativity and Team Adaptivity

Team adaptivity is defined as the degree to which team members cope with and respond effectively to changes that affect their team (Griffin et al., 2007). Team adaptivity captures the capacity of teams to modify their internal structure and functioning in response to external change (Griffin et al., 2007).

To substantiate the hypothesized relationship between team creativity and NPD team adaptivity, this article builds on the notion that creativity is considered as a mechanism of adaptation (Burke et al., 2006; Hülsheger et al., 2009). As Maynard et al. (2015) explained, teams that encounter early creativity will tend to adapt more easily to changes. This positive association can be explained by how creative teams approach adaptive situations (Burke et al., 2006; LePine, 2005). First, because of their broader cognitive scope, creative teams are more likely to process a comprehensive amount of relevant information when they are confronted with a novel element in their environment (Gilson & Shalley, 2004). In that sense, creative teams are better positioned to develop a holistic comprehension of the adaptive demand (Hargadon & Bechky, 2006). As a result, creative teams are thus more likely to consider aspects of the adaptive situation that are important but not usually salient. Second, because creative teams tend to delve into unknown areas to produce a greater number and a larger range of ideas, they are likely to develop a more diverse repertoire of adaptive responses (Paulus, 2000). In other words, teams with high creativity levels have fuller cognitive toolboxes (Richard et al., 2018) from which they can choose many alternative solutions to adapt to changes. Therefore, NPD teams that show creativity are more likely to choose an adaptive response that matches the demands of the adaptive situation. To summarize, as a result of a more complete understanding of the adaptive situation and of a more diverse repertoire of potential adaptive responses, NPD teams characterized by high levels of creativity should be better able to adapt their internal structure and functioning to respond effectively to changes. Based on these arguments, we propose our second research hypothesis:

*H2*: Team creativity is positively related to NPD team adaptivity.

### The Mediating Role of Team Creativity

Taken together, Hypotheses 1 and 2 propose that team PsyCap carries important adaptive benefits for NPD teams that can be explained by members’ creative behaviors. In other words, we argue that shared psychological resources such as team-level hope, efficacy, resilience, and optimism do not directly translate into higher levels of team adaptivity. Rather, we suggest that the possession of these shared psychological resources will predispose members to engage in creative behaviors which, in turn, will make them more likely to adapt successfully to changes that affect their team. As previously discussed, this is consistent with psychological resource theories and with the Input–Throughput–Output model of team adaptation, which explain that psychological resources are likely to enhance adaptive capacities indirectly by promoting behaviors that are conducive of adaptation (Hobfoll, 2002; Van den Heuvel et al., 2014). Overall, previous research provides support for the conceptualization of team creativity as the behavioral mechanism that accounts for the positive influence that team PsyCap exerts on NPD team adaptivity. Therefore, based on our previous arguments, we formulate our third research hypothesis:

*H3*: Team creativity mediates the positive relationship between team psychological capital and NPD team adaptivity.

### The Moderating Role of Outcome Focus

Outcome focus captures the extent to which a team emphasizes performance results and gaining favorable evaluations. Similar to the concept of team performance orientation (Gong et al., 2013), outcome-focused teams prioritize the criteria by which they will be evaluated and the performance levels they have to reach (Bunderson & Sutcliffe, 2003; Woolley, 2009). As such, outcome focus is a shared understanding between team members that performance outcomes (the ends) should take precedence over and constrain process (the means; Woolley, 2009). In that way, outcome-focused teams concentrate their discussions and efforts on instrumental aspects of the task. In the context of creativity-driven projects such as NPD, we argue that a collective focus on outcomes and performance will offset the motivational impact and narrow the broadening effect of team PsyCap, and thus exert a negative influence on the relationship between NPD team PsyCap and team creativity.

First, outcome-focused NPD teams are mostly motivated by how performance will be judged and by their progress toward goal attainment. As a result, ideas of members are likely to be evaluated in terms of their utility for responding to the criteria of evaluation and for attaining desired levels of performance. However, previous research has consistently shown that expectations of evaluation can be detrimental to the creative process (Amabile & Pratt, 2016; Paulus, 2000; Woodman et al., 1993). This is because creative behaviors often seem more distal to goals and because they can be perceived as slowing the team down. As such, a high degree of focus on outcomes is likely to send situational cues that will reduce the willingness of members to use their shared psychological resources to engage in creative behaviors. In addition, this entails that members of outcome-focused NPD teams are less likely to capitalize on the broadening of cognitions that is generated by team PsyCap to propose new and creative ideas that would contribute to the innovativeness and overall quality of their product development. In contrast, under lower levels of outcome focus, team members may feel psychologically safer when proposing novel ideas (Edmondson, 1999; M. A. West, 1990), which will make them more susceptible to take advantage of their shared positive psychological resources to explore and experiment alternative ways of designing their product.

Second, because outcome-focused teams prioritize goals and performance, they tend to frame errors in a negative way (Button et al., 1996). Specifically, members of outcome-focused teams will mostly perceive errors as performance impediments and thus will mainly focus on avoiding failures and reducing risk (Sternberg & Lubart, 1995). However, because creativity is a risk-taking behavior and because creative processes such as NPD are fraught with repeated iterations, errors and reframing are of critical importance (Woodman et al., 1993). As such, considering their preference for avoiding errors, outcome-focused NPD teams are less likely to tap into their pool of shared psychological resources to explore and experiment. On the contrary, NPD teams characterized by low levels of outcome focus are more likely to perceive errors as learning opportunities, which will make them more likely to benefit from the motivational impact and from the broadened cognitive scope that accompanies team PsyCap. To summarize, we argue that the evaluative atmosphere and the negative framing of errors that are likely to accompany outcome-focused NPD teams will offset the motivational impact and narrow the broadening of cognitions that is generated by team PsyCap, and thus weaken its positive influence on team creativity. As such, we propose our fourth research hypothesis:

*H4*: Outcome focus moderates the strength of the relationship between NPD team PsyCap and team creativity, such that this relationship will be stronger under low levels of outcome focus.

### Moderated Mediation Model

Hypothesis 3 states that team creativity mediates the relationship between NPD team PsyCap and team adaptivity. Hypothesis 4 proposes that outcome focus moderates the relationship between NPD team PsyCap and team creativity. When combined, these hypotheses specify a first-stage moderated mediation model that suggests that the indirect effect of NPD team PsyCap on team adaptivity through team creativity is conditional on the level of outcome focus. The fifth hypothesis is formulated based on this moderated mediation model:

*H5*: The indirect effect of team PsyCap on team adaptivity through team creativity is moderated by outcome focus, such that this indirect effect is stronger when the level of outcome focus is low.

## Method

### Participants and Procedures

Research data were gathered from a sample of 1,016 participants grouped into 198 project teams. Participants are graduate and undergraduate students from a Canadian business school who took part in an NPD project management simulation called “Pegasus Simulation” (Aubé et al., 2018). In order to have an adequate sample size, this study was conducted over a three-year period (i.e., nine academic terms). The team formation process was conducted in a way that ensures a degree of demographic and functional diversity in each team. Team size varied from four to six members, men made up 52% of the sample, and the average age of participants was 26 years (*SD* = 5.7 years).

Teams initially receive a business case explaining that they work for a large firm that specializes in the transportation of hazardous materials and that they have been mandated to design and build a vehicle that will transport a container for a petroleum company. Teams had 6.5 hr to develop and build a scale model of their vehicle using a Meccano set (construction game). Teams were fully autonomous in the management of their time and resources. For example, teams could spend their budget to order parts, to hire consultants, and to evaluate the performance of their vehicle on a test track. At the end of the simulation, the vehicle built had to be able to travel two given routes, the second route being more rugged than the first. Teams reached their goal completely when their vehicle was able to travel both paths without stalling or drifting offroad.

The Pegasus simulation is particularly well suited to the study of NPD teams because this simulation was designed to reproduce the main features of a new product development project. For example, teams had to design their vehicle, manage a budget, entertain relationships with diverse stakeholders, and progress in spite of a lack of information. Also, as is the case in real new product development projects, teams were confronted with multiple unforeseen events. These externally imposed events (e.g., stockouts, budget compressions, change in requirements, and reduction in timeframe) were planned in advance and occurred at the same time for each team. Performance depended on the capacity of teams to adapt their internal functioning to these unanticipated changes. Overall, by recreating important features of a team-based new product development project, the Pegasus simulation offers an adequate level of ecological validity.

### Measures

To reduce common method variance, data were collected from two sources and through two methods of evaluation. Team members provided data regarding the psychological capital of their team through a self-report questionnaire administered at the end of the simulation, whereas ratings concerning team creativity, team adaptivity, and outcome focus were provided by one observer, who was a doctoral student blind to the research hypotheses. This individual conducted all observations and was trained beforehand by viewing video recordings of previous simulations that were not part of this data set, assessing inter-coder agreement and resolving misunderstandings with a more experienced member of our research team. The observer assessed each team based on real-time observations done during four 10-min periods. To establish the observation points, the simulation was divided into four equivalent parts. Moreover, to capture meaningful team dynamics and interactions, these observation points were placed right after the announcement of four critical events: first major stockout, change in client requirements, budget compression, and shortened deadline. Considering that the observer had to rate four to six teams per session, there was a small gap between the moments in which each observations point was conducted. In total, the observer thus carried out 40 min of observation per team to judge team creativity, team adaptivity, and outcome focus.

#### Team Psychological Capital

Team psychological capital was measured using the eight-item psychological capital questionnaire by Heled et al. (2016). Sample items include: “*Members of this team confidently contribute to discussions about the team’s strategy*” (efficacy); and “*Members of this team usually take stressful things in stride*” (resilience). Items were rated on a 5-point Likert scale ranging from *not true at all* (1) to *totally true* (5).

#### Team Creativity

Team creativity was evaluated by using the 4-item scale developed by Shin and Zhou (2007). Sample items include: “*How well does this team produce new ideas*”; and “*How useful were those ideas*.” Items were rated on a 7-point scale ranging from *extremely weak* (1) to *excellent* (7).

#### Outcome Focus

Outcome focus was evaluated by using the scale developed by Woolley (2009). Due to low factor loading, one item was removed. As such, the observer rated the amount of attention that teams gave to each of the following three issues on a 5-point scale ranging from *not at all* (1) to *very much* (5): (a) “*what constitutes a successful performance on this task*” (b) “*what criteria will be used for evaluating the final product*”; and (c) “t*he relative importance of the different parts of the task for the final score*.”

#### Team Adaptivity

Team adaptivity was measured using the 3-item scale developed by Griffin et al. (2007). Sample items include: “*Members of this team dealt effectively with changes affecting their team*”; and “*Members of this team responded constructively to changes in the way their team works*.” The observer used a 5-point scale ranging from *not true at all* (1) to *totally true* (5) to measure this variable.

#### Control Variables

Given their potential influence on the present study’s variables, team size and prior task experience were considered as control variables. Team size was included following previous findings that size impacts both team functioning and outcomes (LePine et al., 2008). In the context of this study, NPD teams composed of more members might possess a more diverse composition in terms of functional expertise which, in turn, could contribute to their collective capacity to produce creative ideas and adapt effectively to changes.

Prior task experience was also controlled for given that teams composed of members with previous experience with the Meccano set are better positioned to generate creative ways of constructing their vehicle and adapt effectively to task-related changes. Prior task experience was assessed by asking participants how much experience they had on a scale ranging from *not at all* (1) to *very much* (5) with the Meccano construction game before the start of the simulation.

## Results

### Preliminary Analyses

#### Data Aggregation

Data regarding team PsyCap were collected at the individual level. To justify aggregating these ratings to the team level, it is necessary to demonstrate sufficient interrater agreement and between-group variability. To evaluate interrater agreement, we calculated the *r*_wg_ index (James et al., 1993). Results indicated that the average *r*_wg_ score for team PsyCap was .94, which is deemed an acceptable value (LeBreton & Senter, 2008). To assess between-group variability, we first calculated the intraclass correlation coefficient ICC(1). The usual rule of thumb establishes that an ICC(1) value that exceeds .05 warrants aggregation (Bliese, 2000). Results revealed that the ICC(1) value for team PsyCap was .32, which indicates strong team membership effects. Lastly, to assess if team means were reliably different from one another, we calculated the ICC(2) coefficient. For the ICC(2), values greater than .60 are considered as evidence of significant between-unit variability (Glick, 1985). The ICC(2) value for team PsyCap was .71, which indicated that there was more agreement within teams than between teams. Taken together, these results warrant the aggregation of individual scores to the team level for the team PsyCap construct.

#### Confirmatory Factor Analyses

Because the data about the moderator, the mediator, and the dependent variable were all collected from the same observer, confirmatory factor analyses (CFAs; Amos 27, maximum likelihood estimation) were conducted to establish the validity and distinctiveness of team creativity, outcome focus, and team adaptivity. Overall, goodness-of-fit indices indicated that this intended three-factor model fits the data well (χ^2^[31] = 75, *p* < .001; Tucker–Lewis index (TLI) = 0.95; comparative fit index (CFI) = 0.96; standardized root mean square residual (SRMR) = 0.06; root-mean-square error of approximation (RMSEA) = 0.09). All items were also significantly related to their respective latent constructs (*p* < .001).

In addition, we compared the intended three-factor structure with a range of alternative models. As can be seen in Table 1, results of chi-square difference tests showed that the three-factor model was significantly different than the alternative models in terms of fit. Specifically, the fit of the intended model was significantly better than models in which: (a) outcome focus and team creativity were combined into a single factor (*∆*χ^2^[2] = 106, *p* < .001), (b) team creativity and team adaptivity were combined into a single factor (*∆*χ^2^[2] = 149, *p* < .001), (c) outcome focus and team adaptivity were combined into a single factor (*∆*χ^2^[2] = 302, *p* < .001), and (d) all items were gathered within one latent variable (*∆*χ^2^[3] = 254, *p* < .001). Overall, these results indicated that the three variables measured by the observer were distinct and thus appropriate for inclusion in subsequent analyses.

**Table 1.** Measurement Model Comparisons.

Models	χ^2^ (*df*)	*TLI*	*CFI*	*RMSEA*	*SRMR*	χ^2^_diff_	*df*_diff_
Intended model, three factors	75 (31)	0.95	0.96	0.085	0.059		
Model A, two factors^a^	181 (33)	0.83	0.88	0.151	0.115	106	2***
Model B, two factors^b^	224 (33)	0.78	0.84	0.172	0.101	149	2***
Model C, two factors^c^	377 (33)	0.61	0.71	0.230	0.237	302	2***
Model D, one factor^d^	329 (34)	0.67	0.75	0.210	0.138	254	3***

*Note. n* = 198. χ^2^ = chi-square discrepancy; *df* = degrees of freedom; IFI = incremental fit index; TLI = Tucker–Lewis index; CFI = comparative fit index, GFI = goodness of fit index; RMSEA = root mean square error of approximation; SRMR = standardized root mean square residual; χ^2^_diff_ = difference in chi-square; *df*_diff_ = difference in degrees of freedom.

a Outcome focus and team creativity combined into a single factor, compared to the three-factor model.

b Team creativity and team adaptivity combined into a single factor; compared to the three-factor model.

c Outcome focus and team adaptivity combined into a single factor; compared to the three-factor model.

d Single factor model: all items combined into a single latent factor; compared to the three-factor model.

*** *p* < .001.

#### Common Method Variance

Before proceeding to hypotheses testing, we also wanted to assess the degree to which common method bias was a pervasive issue in our study. Specifically, we wanted to make sure that the variance of the three variables assessed by the observer was true variance and not variance that is attributable to the common measurement method. To do so, we first conducted Harman’s single factor test (Harman, 1960). This technique uses exploratory factor analysis in which variables are constrained so that there is no rotation. Common method bias is assumed to exist if (1) a single factor emerges, or (2) a first factor explains more than 50% of the variance (Podsakoff et al., 2003). Results of principal component analysis in SPSS revealed three distinct factors that accounted for 74% of the total variance. Moreover, the first unrotated factor captured 43% of the variance in the data. Therefore, no single factor emerged, and the first factor did not account for the majority of the covariance among the measures. We also used the technique of Bagozzi et al. (1991) for assessing the impact of common method variance through latent variable correlation. They explained that common method bias is evident when a substantial correlation (*r* ≥ .90) is found among principal constructs. In that regard, correlation analyses indicated that the strongest association was between team creativity and team adaptivity (*r* = .52). For these reasons, we believe that common method effects are unlikely to be a major issue in our data. Descriptive statistics (means and standard deviations), reliability estimates, and correlations for the study variables are presented in Table 2.

**Table 2.** Descriptive Statistics, Correlations, and Reliability Estimates.

Variables	Mean	*SD*	1	2	3	4	5
1. Team psychological capital	4.14	0.39	(.85)				
2. Team creativity	4.59	0.92	.28**	(.93)			
3. Team adaptivity	3.42	0.72	.21**	.52**	(.78)		
4. Outcome focus	2.13	0.66	.06	.16*	−.02	(.72)	
5. Team size	5.13	0.74	.06	.10	.07	.08	N/A
6. Prior task experience	1.64	0.50	.26**	.13	.07	.12	.20

*Note. n* = 198 teams. *SD* = standard deviation. Reliability estimates (Cronbach’s alphas) are in parentheses.

* *p* < .05, two-tailed. **p < .01, two-tailed.

### Hypotheses Testing

We tested our study hypotheses in three interrelated steps. First, we examined the mediational pathway comprised of team PsyCap, of team creativity, and of team adaptivity (Hypotheses 1–3). Second, we investigated the moderating effect of outcome focus (Hypothesis 4). To test these four hypotheses, we used a path analytic procedure with the Amos 27 software and the maximum likelihood method. Third, we assessed the indirect effect of team PsyCap on team adaptivity at different values of outcome focus (Hypothesis 5). To do so, we relied on the SPSS macro PROCESS (model 7; Hayes & Preacher, 2013).

#### Test of Mediation

Table 3 presents the results for Hypotheses 1 to 3. In support of Hypothesis 1, NPD team PsyCap was found to be positively related to team creativity (β = 0.63, *t* = 3.78, *p* < .001). Also, in support of Hypothesis 2, the path estimate for the relationship between team creativity and team adaptivity was significant (β = 0.39, *t* = 7.85, *p* < .001).

**Table 3.** Path Analysis Results for Mediation.

	Team creativity				Team adaptivity			
	β	*SE*	*t*	*p*	β	*SE*	*t*	*p*
Team size	0.09	0.09	1.09	.276	0.02	0.06	0.26	.794
Prior task experience	0.08	0.13	0.63	.528	−0.03	0.09	0.29	.770
Team PsyCap	0.63	0.17	3.78	.001	0.13	0.12	1.06	.290
Team creativity					0.39	0.05	7.85	.001
*R* ^2^	.09				.27			
	*M*		*SE*		LL 95% CI		UL 95% CI	
	Bootstrap results for indirect effect							
Effect	0.25	0.07	0.13		0.41			

*Note. n* = 198 teams. Unstandardized regression coefficients are reported. Bootstrap sample size = 10,000. LL = lower limit; CI = confidence interval; UL = upper limit.

Hypothesis 3 states that the relationship between NPD team PsyCap and team adaptivity is mediated by team creativity. This indirect effect was assessed using the bootstrapping strategy as recommended by Preacher and Hayes (2008). Based on a 10,000 bootstrap samples, results revealed that the indirect effect of NPD team PsyCap on team adaptivity through team creativity was significant (Indirect effect = 0.25, *SE* = 0.07, bias-corrected 95% confidence interval (CI) = [0.13, 0.41]). Moreover, when team creativity was entered in the regression equations, the relationship between NPD team PsyCap and team adaptivity was rendered non-significant (β = 0.13, *t* = 1.06, *p* = .29, 95% CI = [−0.11, 0.37]), therefore confirming Hypothesis 3. Lastly, considering that the simulation was the first time team members worked together, we wanted to test an alternative mediational pathway in which team PsyCap was the outcome rather than the input. Specifically, we compared our intended mediation model with a reverse set of relationships in which the creative behaviors of team members enhance their adaptivity which, in turn, leads to the development of team PsyCap. Results showed that the intended mediation model (χ^2^[1] = 1.11, *p* = .29; TLI = 0.98; CFI = 0.99; SRMR = 0.02; RMSEA = 0.02) had a much better fit than the reverse model (χ^2^[1] = 7.24, *p* < .01; TLI = 0.33; CFI = 0.93; SRMR = 0.04; RMSEA = 0.18). In addition, results indicated that the intended mediation model had a lower Akaike Information Criterion (29) than the alternative model (35). Taken together, these results demonstrate the superiority of our mediation model when compared to an alternative pathway in which team PsyCap is the outcome.

#### Test of Moderation

**Figure 2.**
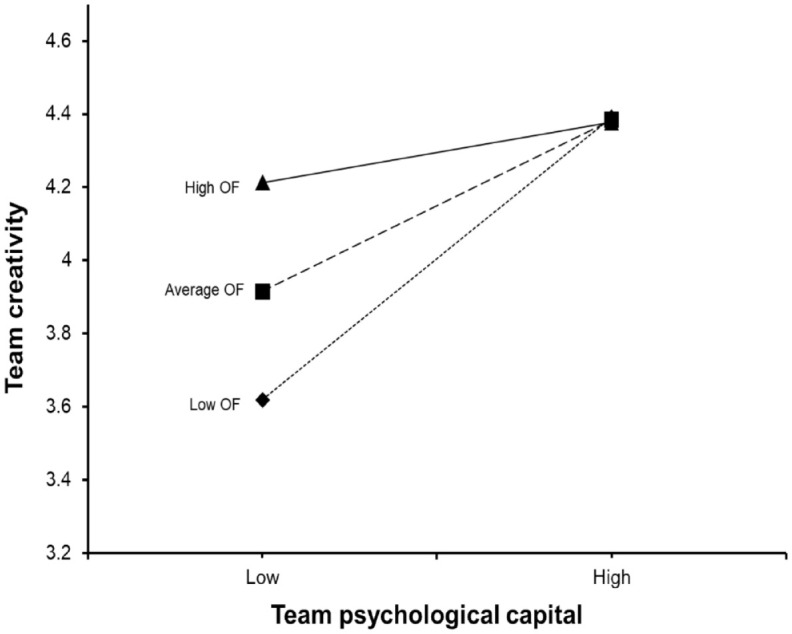
Moderating effect of outcome focus. *Note*. OF = outcome focus.

Hypothesis 4 suggests that outcome focus will negatively moderate the positive relationship between NPD team PsyCap and team creativity. To test this hypothesis, we created a cross-product interaction term involving team PsyCap and outcome focus. Results revealed that the interaction term had a significant path estimate (β = −0.59, *t* = 2.77, *p* < .01) and that its inclusion in the model explained an additional 5% of the team creativity variance, which provides support for the moderating effect of outcome focus. To fully support Hypothesis 4, the form of this interaction should conform to the hypothesized pattern. Based on recommendations by Cohen et al. (2003), the moderating effect was interpreted by plotting the regression equations in relation to three levels of outcome focus, namely the mean, one standard deviation below the mean, and one standard deviation above the mean (see Figure 2). In line with our expectations, the slope of the relationship between team PsyCap and team creativity was significant for NPD teams with low levels (β = 0.99, *t* = 4.73, *p* < .001) and average levels (β = 0.55, *t* = 3.38, *p* < .001) of outcome focus, whereas the slope of the relationship was non-significant for NPD teams with high levels of outcome focus (β = 0.21, *t* = 0.96, *p* = .34). Hypothesis 4 is thus supported by the results of simple slopes analyses and the results depicted in Figure 2, which means that outcome focus negatively moderates the relationship between NPD team PsyCap and team creativity.

However, upon closer inspection, results reveal the presence of a substituting effect. A substituting effect occurs when the predictor and moderator both exert positive effects on the criterion, and the conditional *X–Y* relationship is weakened as *Z* increases (Gardner et al., 2017). Substituting effects imply the presence of antagonistic forces where both the independent and moderating variables positively influence the criterion but where neither adds value beyond the other (Cohen et al., 2003). In the present study, findings show that while outcome focus weakens the positive effect that team PsyCap exerts on team creativity, it is positively related to team creativity (β = 0.38, *t* = 3.61, *p* < .001). Moreover, as shown in Figure 2, NPD teams characterized by high levels of outcome focus and low levels of PsyCap were able to attain similar levels of creativity than teams with high PsyCap and low outcome focus. This finding illustrates how outcome focus acts as a substitute to team PsyCap in enhancing team creativity.

#### Test of Moderated Mediation

Overall, goodness-of-fit indices revealed that the full moderated mediation model fits the data well: χ^2^[2] = 4, *p* = .11; TLI = 0.76; CFI = 0.98; SRMR = 0.02; RMSEA = 0.08). Based on 10,000 bootstrap samples, the value of the index of moderated mediation was −0.23 (95% CI = [−0.42, −0.08]), which confirms the statistical significance of the proposed model. We also examined the conditional indirect effect of NPD team PsyCap on team adaptivity through team creativity at three values of outcome focus (see bottom of Table 4): the mean, one standard deviation above the mean, and one standard deviation below the mean. Results indicated that the estimate of conditional indirect effect was not significant at high levels of team outcome focus (Indirect effect = 0.08, 95% CI = [−0.07, 0.23]). However, this estimate was significant at average levels (Indirect effect = 0.24, 95% CI = [0.12, 0.36]) and at low levels of outcome focus (Indirect effect = 0.39, 95% CI = [0.22, 0.57]). These results mean that the indirect effect increases in magnitude as the level of outcome focus decreases.

**Table 4.** Results for Conditional Indirect Effect.

Predictor	β	*SE*	*t*	*p*	LL 95% CI	UL 95% CI
*Team creativity*						
Constant	4.15	0.45	9.14	.001		
Team size	0.07	0.08	0.78	.435		
Prior task experience	0.07	0.13	0.54	.588		
Team PsyCap	0.60	0.16	3.69	.001		
Outcome focus	0.22	0.09	2.32	.020		
Team PsyCap × Outcome focus	−0.59	0.22	2.77	.006		
*R* ^2^	0.14					
*Team adaptivity*						
Constant	1.58	0.38	4.13	.001		
Team creativity	0.39	0.05	7.85	.001		
*R* ^2^	.27					
	Boot indirect effect	Boot *SE*		Boot *p*		
Outcome focus	Conditional indirect effect at outcome focus = *M* ± 1 *SD*					
−1 *SD* (−0.66)	0.39	0.09		.001	0.22	0.57
*M* (0)	0.24	0.06		.001	0.12	0.36
+1 *SD* (0.66)	0.08	0.08		.222	−0.07	0.23

*Note. n* = 198 teams. Unstandardized regression coefficients are reported. Bootstrap sample size = 10,000.

Pairwise contrast analyses confirmed these results by demonstrating that the indirect effect of NPD team PsyCap on team adaptivity was significantly different at high and low levels of outcome focus (95% CI = [−0.55, −0.11]). Overall, these results suggest that team PsyCap only exerts an indirect effect on team adaptivity when NPD teams exhibit moderate or low levels of focus on outcomes. Therefore, Hypothesis 5 is supported such that the indirect and positive effect of NPD team PsyCap on team adaptivity via team creativity depends on the level of outcome focus.

## Discussion

The purpose of the present study was to investigate team-level variables that contribute to the adaptability of NPD teams. Drawing from psychological resource theories, we aimed to uncover how and under which conditions team PsyCap promotes the adaptivity of NPD teams. We found support for our model which proposed that team PsyCap positively influences NPD team adaptivity through members’ engagement in creative behaviors. In addition, we found that outcome focus moderates the relationship between NPD team PsyCap and team creativity, such that this relationship is only present at low and moderate levels of outcome focus but not at higher levels. However, we also uncovered that although outcome focus nullifies the positive effect of team PsyCap on team creativity, it serves as a substitute to team PsyCao in enhancing team creativity. Overall, results of the present contribute to our understanding of the influence of psychological resources in the context of NPD teams. Below, we discuss the theoretical contributions and managerial implications of our findings.

### Theoretical Contributions

Project management researchers have recently called for further investigation into the adaptability of NPD teams (Durmusoglu & Calantone, 2022). Similarly, in their review of 15 years of team adaptation research, Maynard et al. (2015) concluded that the lack of empirical studies that explore the antecedents of team adaptation is striking. In light of these shortcomings, the present study’s focus on team-level predictors of NPD team adaptivity makes several important contributions.

First, results show that teams who possess high levels of collective hope, efficacy, resilience, and optimism are better able to modify their internal structure and functioning in order to respond to changes. This finding is even more relevant in the context of NPD teams because the complex and dynamic nature of their task makes psychological factors and adaptation key aspects of project success (Cooper, 2019; Stock et al., 2021). As such, by uncovering the previously unstudied relationship between team PsyCap and team adaptivity, this study develops our understanding of the important role that shared positive psychological resources play in the adaptive process of NPD teams. In addition, this result provides evidence of the applicability of the propositions put forth by psychological resource theories to the team level of analysis. As previously mentioned, these theories explain that individuals tap into their pool of psychological resources in order to adapt to changes. By validating the isomorphic properties of this association, this study elevates psychological resource theories to the team level and shows that positive psychological resources are also important factors for the effective adaptation of teams.

Second, our results indicate that team creativity also plays a central role in the adaptive process of NPD teams. Specifically, we demonstrate that members’ engagement in creative behaviors is an important adaptive mechanism that serves to explain how shared psychological resources contribute to NPD team adaptivity. Importantly, this finding helps clarify the nature of the relationship between team creativity and team adaptation in an NPD context. As Maynard et al. (2015) explained, team creativity is as likely to be a by-product as an underlying process of team adaptation. Findings of this study suggest that it is through the creative behaviors of members that NPD teams are able to adapt effectively, thus providing tentative evidence of the precedence of team creativity. Furthermore, the finding that team creativity mediates the positive association between NPD team PsyCap and team adaptivity also contributes to the team creativity scholarship. More precisely, despite the collective nature of many creative endeavors, research on creativity has focused on the individual (Hargadon & Bechky, 2006). As a result, less energy has been devoted to identifying factors that enhance the creativity of teams (M. A. West, 2002). Thus, our finding that team PsyCap enhances the creativity of NPD teams responds to the lack of research exploring the antecedents of team creativity and aligns well with Amabile and Pratt’s (2016) call to explore how psychological factors influence creativity.

Third, another contribution of this study resides in the finding that the creative benefits of team PsyCap, and through this its positive effect on team adaptivity, are contingent on the level of outcome focus. Specifically, we show that outcome-focused NPD teams tend to adapt less effectively because they are less likely to take advantage of their psychological resources to propose creative ideas. This indicates that although team PsyCap is an important predictor of team creativity in NPD settings, the degree of outcome focus is also a critical aspect to consider as it dampens the positive influence that shared positive psychological resources exert on the collective engagement in creative behaviors and ultimately on the capacity of NPD teams to adapt effectively. However, our results revealed that although outcome focus exerts a negative influence on the indirect relationship between team PsyCap and team adaptivity through team creativity, it is positively related to team creativity. This means that while a high degree of outcome focus nullifies the creative and adaptive benefits of shared psychological resources, this type of team goal orientation still promotes members’ engagement in creative behaviors. In other words, while outcome-focused NPD teams might not be able to reap the creative benefits of their psychological resources, their high performance drive and their willingness to receive positive evaluations will motivate members to propose novel and creative ways to develop their product (Gong et al., 2013). This interesting finding highlights that outcome focus serves as a substitute to team PsyCap in enhancing team creativity. This means that because their respective effects on team creativity cancel each other out, team PsyCap and outcome focus are alternative and antagonistic paths to team creativity. Considering that the creative and adaptive consequences of a collective focus on outcomes and performance are not well understood (Caniëls et al., 2019; Christian et al., 2017), these findings provide evidence that may serve to clarify these inconsistent results. For example, some studies found that teams emphasizing outcomes and performance tend to exhibit fewer creative behaviors and adapt less effectively (Bunderson & Sutcliffe, 2003; LePine, 2005; Porter, 2005). As LePine (2005) explained, teams that emphasize outcomes and performance tend to approach adaptive situations in terms of “how the disruption would affect progress of performance and less about possible actions the team should take in order to cope with the disruption itself” (p. 1163). In contrast, other studies report that a collective focus on outcomes and performance positively influences the creative and adaptive performance of teams (Porter et al., 2010; Woolley, 2009). Specifically, some studies have shown that a high degree of focus on outcomes and performance motivates members to share information and resources, which allows them to identify actions at higher-levels and adapt more effectively (Gong et al., 2013; Woolley, 2009). In relation to these inconsistent findings, our results highlight the complex relationship that exists between outcome focus and team creativity.

Lastly, results of this study also contribute to the development of the nomological network of team PsyCap. More precisely, while previous research has mainly focused on confirming the positive effect that team PsyCap exerts on multiple dimensions of team performance, this study extends current knowledge by exploring the previously unstudied relationship between team PsyCap and team adaptivity. Moreover, research is just beginning to scratch the surface of the mediators that intervene in the relationships between team PsyCap and its outcomes (Luthans & Youssef-Morgan, 2017). The present study addresses this need by demonstrating that it is as a result of the collective engagement in creative behaviors that team PsyCap enhances the adaptivity of NPD teams. Lastly, our work uncovered a novel boundary condition influencing the impact of team PsyCap. In doing so, the present study responds to the lack of research investigating the moderators team PsyCap and its outcomes (Newman et al., 2014).

### Managerial Implications

Because NPD teams operate in dynamic and uncertain environments, a better understanding of the factors that promote their adaptive capacities carries important implications for managers. Specifically, our results highlight that managers of NPD projects should promote and foster the psychological resources of their team. To do so, previous research informs us that promotive actions may include adopting a humble or transformational style of leadership (Rebelo et al., 2018; Rego et al., 2017), sharing leadership roles and responsibilities with team members (Wu & Chen, 2018), showing confidence in the capacities and chances of success of the team, and creating a psychologically safe team climate where learning behaviors are encouraged (Gonçalves & Brandão, 2017).

NPD projects also require that team members engage in creative behaviors. In that regard, our results indicate that when managers of NPD projects want to harness the creative benefits of the psychological resources of their team, they should be very careful that performance outcomes are not overemphasized and come to take precedence over and constrain the creative process. In order to do so, managers should frame NPD as a learning process where risk-taking behaviors are encouraged and where failure and errors are seen as learning opportunities. Moreover, in the early stages of the NPD project, leaders should encourage team members to brainstorm and to generate as many ideas as they can, rather than focusing straight away on performance outcomes and expectations. In other words, to take advantage of the creative benefits that the shared psychological resources of their team generate, team managers should emphasize that the process (how) is as important as the results (what).

Moreover, our results also suggest that when NPD teams exhibit low levels of hope, efficacy, resilience, and optimism, the development of a collective focus on outcomes and performance can act as a substitute to team PsyCap and be beneficial to team creativity. As such, mangers of NPD projects should be aware of the antagonistic relationship that exists between both these predictors of team creativity. For example, when their team is going through a rough patch in the development of their new product and is showing signs of low PsyCap, managers can continue to foster the creative behaviors of members by promoting and reorienting the team towards its final outcome and the criteria by which the product will be evaluated. In doing so, this restatement and refocus on outcomes and performance can infuse the team with the energy needed to engage in creative behaviors.

### Limitations and Directions for Future Research

As is the case with most research designs, some methodological limitations are present in this study. Specifically, although the duration of the simulation was 6.5 hr, this research is still cross-sectional in nature. As such, we cannot make definitive statements about the causality between team PsyCap, team creativity, and team adaptivity. However, we draw on our theoretical arguments and on the established Input–Throughput–Output model of team adaptation to suggest that our data support a model in which NPD team PsyCap increases team creativity, and though this team adaptivity. Moreover, a reverse mediational model in which team creativity led to the development of team PsyCap through enhanced task adaptivity was tested and found to have a worst fit than the proposed model. Nonetheless, future research investigating the relationship between team PsyCap and team adaptation would benefit from using multistage longitudinal designs where variables are measured at multiple points in time.

Second, because this study was conducted by means of a simulation and with a sample of student teams, the generalizability of our results may be limited. For example, a secure environment like the one that was used in this study may not replicate the adaptive demands and the levels of perceived importance of outcomes that are faced by NPD project teams working in organizations. However, previous research has shown that correlations between effect sizes obtained in laboratory and field settings exceed .70, which suggests that the use of a student sample may not severely affect generalizability (C. A. Anderson et al., 1999). Nonetheless, the external validity of the findings of this study should be assessed with caution until the results have been replicated in organizational settings.

Beyond addressing the limitations of this study, it would be worthwhile to consider other interesting avenues for future research. For example, our model is not exhaustive in terms of considering the range of mediators that might intervene in the relationship between NPD team PsyCap and team adaptability. Therefore, future research investigating the relationship between psychological resources and team adaptation would do well to include other types of mediators. Moreover, considering the scarcity of studies that investigate the boundary conditions of team PsyCap, we call on future research to explore other moderators that might influence the effects of shared positive psychological resources in the context of NPD teams. For example, it would be interesting to capture how features of the organizational context influence the relationship between team PsyCap and team adaptation. Lastly, this study provided evidence of the negative influence that a collective focus on outcomes exerts on the relationship between NPD team PsyCap and team creativity. Considering the inconsistent results related to the concept of outcome focus, future research should validate if the results of this study translate to other types of projects and in different types of teams

## Conclusion

In closing, we examined team-level variables that promote the adaptivity of NPD teams. Specifically, we conducted an examination of the mediating and moderating mechanisms operating in the relationship between NPD team PsyCap and team adaptivity. Results demonstrate that NPD team PsyCap has the potential to generate team creativity and through this team adaptivity, contingent on the level of outcome focus. Taken together, these findings provide an important contribution to our understanding of how and under which conditions shared psychological resources promote the capacity of NPD teams to adapt to changes.
